# Current Trends in Advanced Alginate-Based Wound Dressings for Chronic Wounds

**DOI:** 10.3390/jpm11090890

**Published:** 2021-09-07

**Authors:** Andreea Barbu, Bogdan Neamtu, Marius Zăhan, Gabriela Mariana Iancu, Ciprian Bacila, Vioara Mireșan

**Affiliations:** 1 Pediatric Research Department, Pediatric Clinical Hospital Sibiu, 550166 Sibiu, Romania; 2 Faculty of Animal Science and Biotechnologies, University of Agricultural Sciences and Veterinary Medicine Cluj-Napoca, 400372 Cluj-Napoca, Romania; mzahan@usamvcluj.ro (M.Z.); vioara.miresan@usamvcluj.ro (V.M.); 3 Faculty of Medicine, “Lucian Blaga” University of Sibiu, 550169 Sibiu, Romania; gabriela.iancu@ulbsibiu.ro (G.M.I.); ciprian.bacila@ulbsibiu.ro (C.B.); 4 Faculty of Engineering, “Lucian Blaga” University of Sibiu, 550025 Sibiu, Romania; 5 Dermatology Clinic, County Clinical Emergency Hospital, 550245 Sibiu, Romania

**Keywords:** alginate, biomaterial, dressing, fibers, hydrogel, nanofibers, commercially available, wound care, wound healing

## Abstract

Chronic wounds represent a major public health issue, with an extremely high cost worldwide. In healthy individuals, the wound healing process takes place in different stages: inflammation, cell proliferation (fibroblasts and keratinocytes of the dermis), and finally remodeling of the extracellular matrix (equilibrium between metalloproteinases and their inhibitors). In chronic wounds, the chronic inflammation favors exudate persistence and bacterial film has a special importance in the dynamics of chronic inflammation in wounds that do not heal. Recent advances in biopolymer-based materials for wound healing highlight the performance of specific alginate forms. An ideal wound dressing should be adherent to the wound surface and not to the wound bed, it should also be non-antigenic, biocompatible, semi-permeable, biodegradable, elastic but resistant, and cost-effective. It has to give protection against bacterial, infectious, mechanical, and thermal agents, to modulate the level of wound moisture, and to entrap and deliver drugs or other molecules This paper explores the roles of alginates in advanced wound-dressing forms with a particular emphasis on hydrogels, nanofibers networks, 3D-scaffolds or sponges entrapping fibroblasts, keratinocytes, or drugs to be released on the wound-bed. The latest research reports are presented and supported with in vitro and in vivo studies from the current literature.

## 1. Introduction

Chronic wounds represent a major public health issue, with an extremely high cost worldwide. In the USA, chronic wounds affect 1% of the total population, in Europe the incidence is 4 million cases per year which implies a rate of about 0.8%, in Australia 0.86%, in China 0.8–1%, and in India 0.6–1% [1,2]. In healthy individuals, the wound healing process takes place in different stages: inflammation, cell proliferation (fibroblasts and keratinocytes of the dermis), and finally remodeling of the extracellular matrix (equilibrium between metalloproteinases and their inhibitors). In the case of chronic wounds, a chronic inflammatory status is noted, so it takes a longer time to reach the cell proliferation and remodeling (healing) phases. Chronic inflammation favors exudate persistence. The bacterial film has a special importance in the dynamics of chronic inflammation in wounds that do not heal. Clinical studies showed that over 60% of chronic wounds presented a biofilm. Current research envisages advanced wound-dressings to address these disadvantages. These wound-dressings should act by removing the biofilm pathogenic bacteria and modulating the inflammation. Many in vitro and in vivo studies centered upon creating new or better biopolymer-based materials for wound healing in recent years. An ideal wound dressing should adhere to the wound surface and not to the wound bed, it should also be non-antigenic, biocompatible, semi-permeable, biodegradable, elastic but resistant, and cost-effective. It has to give protection against bacterial, infectious, mechanical, and thermal agents, to modulate the level of wound moisture, and to entrap and deliver drugs or other molecules [3,4,5]. Alginate, chitosan, collagen, and cellulose are the most used biomaterials for wound-dressing products [3,6,7,8,9,10]. Of these, alginate is by far the most commonly biomaterial among other bioproducts with wound healing properties [3,6,7].

Because of its hydrophilic nature, alginate is capable to take multiple forms [11,12,13,14] (beads, blends, dressings, electrospun scaffolds, flexible fibers, films, foams, gels, hydrogels, injections, microparticles, microspheres, nanoparticles, polyelectrolyte complex, powders, ropes, sheets, sponges) that could be applied on post-traumatic wounds or exuding wounds (ulcers) while decreasing contamination [15,16,17,18], either as a stand-alone biomaterial, or in various combinations.

This review focuses on the roles of alginates in advanced wound-dressing forms with a particular emphasis on hydrogels, nanofibers networks, 3D-scaffolds or sponges entrapping fibroblasts, keratinocytes, or drugs to be released on the wound-bed. The latest research reports are presented and supported with in vitro and in vivo studies from the current literature.

## 2. Chronic Wounds Mechanisms and Alginates Roles

Wound healing mechanisms involve multiple cellular events, while also being related to the biodynamic of the bacterial film on the wound surface. Inflammation occurs as a result of the inflammatory response of keratinocytes (at the edge of the wound), cytokines, and growth factors during thermal and cellular processes. The cells involved are leukocytes and fibroblasts [19,20,21]. Leukocytes (polymorphonuclear leukocytes-PMN, macrophages, lymphocytes) secrete biomarkers such as IL-1, IL-6, TNF-α, with role for the maintenance of inflammation. Platelets, epithelial cells, endothelial cells, and macrophages secrete growth factors, PDGF (platelets derived growth factors), TGF-β(tumoral growth factor-β), β-FGF(fibroblast growth factor-beta), VEGF-(vascular endothelial growth factor, hypoxia-induced), KGF (keratinocytes growth factors), metalloproteinases-MMPs, and their inhibitors—TIMPs. More than 20 types of matrix metalloproteins have been described to be involved in extracellular matrix (ECM) proliferation [22,23,24].

A fibroblast’s function is to remodel the extracellular matrix and to secrete growth factors. Proliferation is the most critical stage, since the ECM is formed, and the collagen synthesis, reepithelization, and angiogenesis processes begin [25]. Remodeling is represented by the moment when collagen reshapes, the vessels mature and regress from the injured area. Eventually, the reepithelization process takes place [26]. 

A mechanism implicated in unhealing of wounds seems to point out the fact that fibroblasts are unresponsive to growth factors and cytokines. In patients with chronic wounds, increased levels of IL-1 β, IL-6, TNF-α, and an abnormally high ratio MMPs/TIMPs have been found, as demonstrated by computational models, as well. The liquid in chronic wound with its cytokinic composition seems to inhibit the proliferation of dermal fibroblasts by their arrest in the G0/G1 cell cycle by activating an intracellular molecular pathway mediated by Ras protein [27]: (1) High Mobility Group Box Protein 1 (HMGB1) and the analogues involved in wound repair; (2) cell growth mechanisms regulating given by Ras protein. The study of cell matrix and of the ration between metalloproteinases/their inhibitors seems to have a crucial importance in understanding the chronic wounds physiopathology.

Alginates were proved to exhibit: (1) anti-microbial (Gram-positive—Staphylococcus, *Bacillus cereus*; Gram-negative—*E. coli*, *Pseudomonas aeruginosa*, and *Acinetobacter* spp. [9,28,29]); (2) antifungal—*Candida albicans* [9,30,31]; (3) antiviral—*Herpeviridae*, *Rhabdoviridae*, *Flaviviridae*, and *Togaviridae*, due to sulfated polymeric chain [9,32]; (4) anti-anaphylactic; (5) anti-inflammatory, immuno-modulatory by induction of nitric-oxide (NO), reactive oxygen species (ROS), TNF-α, NF-KB release from macrophages, the MAPK signaling pathway; (6) antioxidant; (7) hemostatic by platelets activation and thrombin clot generation; (8) regenerative/angiogenetic properties [28,29,32,33,34,35,36]. Infection is one of the leading causes for a wound to become chronic [34,37] thus making alginate a good candidate when discussing its possible use as a wound dressing especially in hydrogel forms or more advanced solutions such as electrospun nanofibers networks, 3D-scaffolds and sponges entrapping fibroblasts, keratinocytes, or drugs to be released on the wound-bed.

## 3. Alginate Physical Properties

**Figure 1:**
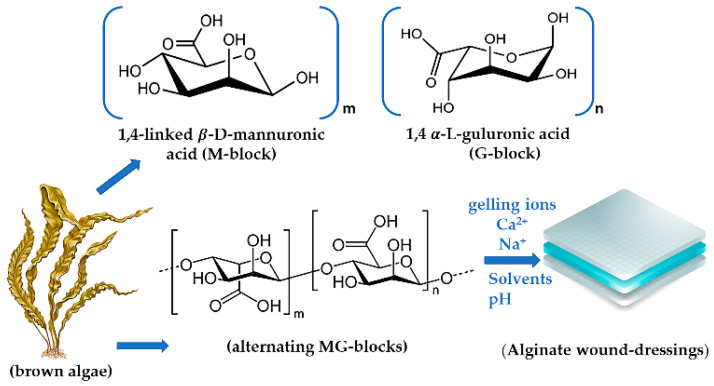
Alginates’ blocks in the polymeric chain.

Alginates (ALG), are linear water soluble high swelling natural anionic polysaccharides obtained from brown algae cell walls and from some bacteria strains such as *Pseudomonas* or *Azotobacter* [6,38,39,40]. They are biopolymers consisting of 1,4-linked *β*-D-mannuronic acid (M) and 1,4 *α*-L-guluronic acid (G) monomers [9,30,39,41]. These monomers are grouped in block-like patterns which can be heterogenous (MG) or homogenous (poly-M, poly-G) (Figure 1). When it comes to terminology, alginate usually refer to alginic acid, and its derivatives [6,9,32,42]. To become water soluble for viscous solutions alginic acid should be converted into ALG esters and monovalent salts like sodium alginate or calcium alginate. The viscosity of sodium alginate aqueous solution (1% *w*/*v*) for example, is highly dynamic ranging between 20 and 400 mPa·s at 20 °C. By tuning the ALG concentration viscosity and other physicochemical properties are influenced [9,43].

The parameters to modulate ALG’s solubility are represented by structure, the carboxylic groups states (protonated/deprotonated), ionic strength, concentration, temperature, the amount of the ‘gelling ions’ such as Ca^2+^ and Na^+^, the solvents, and pH. At a pKa under 3.28–3.65 the solubility is highly affected and the polymer precipitates [9,32]. ALG’s solubility also changes when long alkyl or aromatic groups are attached to their backbone. Then, the presence of protonated carboxylic groups in ALG’s structure comes with the loss of water or any other solvent solubility. Environmental pH also influences ALG mucoadhesive capacity where the polymers carboxyl groups bind with mucin, and if it is higher, the carboxylic groups become deprotonated [9,15,32,42,44].

The gelling ions trigger a cross-link process of the ALG chains and eventually the gelation process [9,32]. Modulating the G-blocks, M-Blocks, or MG-block concentration in the technological process, different gel patterns can be obtained: stiffer, elastic, or flexible [9,32]. When the alginate is fully crosslinked, the gel will be more rigid, with a higher Young’s modulus and lower elongation which affects its tensile strength. The higher the Ca^2+^ concentration the better water resistance and swelling behavior is observed, while in thin films more translucent and clear behavior was noticed [9].

With an impressive swelling capacity (20 times their own weight) ALG weakly jellify in the wound environment, providing moisture and stimulating epidermis regeneration [6,9,40]. ALG are acknowledged to have an excellent biocompatibility and it seems that the adverse events were related to the alginate’s (unobserved) impurities that were added unintentionally in the wound-dressings [9,15,42]. The most used alginate types in wound healing studies are the calcium and the sodium alginate, depending on the wound type or the desired dressing form. The physicochemical properties are correlated with the amount of ALG, more ALG will lead to the viscosity and the bead size to increase [43,45,46]. The used concentration of ALG varies from 0.001% *w*/*v* to 95% depending on the dressing type [47,48,49].

The ALG wound dressings have the ability of exchanging the ‘gelling ions’ with the wound fluids with a direct application in infected wounds. For example, calcium alginate makes a reliable non-woven wound dressing with the ability to exchange Na^+^ in exuding or infected wounds. Consequently, this wound dressing type does not adhere to the wound-bed and the removal is painless. The new formed tissue will not be affected by washing away the alginate fibers. Moreover, there is a self-adherence process in the peri-wound area with a good cover of the affected area [9,15].

In the case of sodium alginate salts, only the water solubility is maintained. It dissolves completely in water but not in organic solvents. Nevertheless, sodium alginate has better gel-forming characteristics [9]. To date, at pH of 1.2 spray-dried particles of sodium alginate consisting of hydrophilic matrix controlled-release form, have a longer release time for the entrapped drugs, forming gels in aqueous media. The speed and the drugs’ absorption rate depend of the wound pH and drug type, but also on the solubility of the alginate salt [9,50]. Some authors state that an alginate-based dressing should be changed every week or when the gel loses its viscos properties [51].

## 4. Alginate-Based Hydrogels for Wound Healing

**Figure 2:**
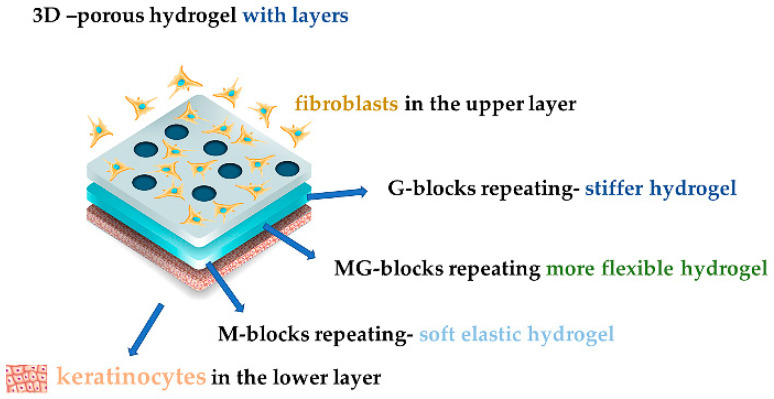
A 3D model of porous hydrogel with fibroblasts and keratinocytes.

One of the most promising alginate forms being used in helping wound healing is the hydrogel because it keeps the moisture and absorbs the excessive exudate, it reduces local pain because it has a cooling effect, it does not adhere to the wound bed and it can hold active compounds such as various drugs, signaling molecules, or stem cells. Their disadvantages are their price and their mechanical instability [52,53]. Their structure influences the obtained gel. Repeating M-blocks have a better water retaining ability that transforms into a softer and more elastic gel, whereas repeating G-blocks will give gels a good mechanical resistance, but they will be stiff, and more MG-blocks will lead to a more flexible gel (Figure 2). ALG rich in M-blocks makes soft flexible gels, while ALG in rich G-blocks make firm gels after they absorb wound secretions [11,23,32,42,49,54]. ALG with high G content reveals interesting in situ gel formation properties with superior results after using a gel instead of a solution for ocular drug delivery, being conditioned by pH and temperature [32,55,56]. An oxidized alginate and borax hydrogel dressing obtained directly in situ with a WVTR (water vapor transmission rate) of 2686 ± 124 g/m^2^/day, was applied on rats, proving that the antiseptic properties of borax helped completely heal the wound within two weeks [57].

If the gelation rate is slow, the gel will be uniform and will have a good mechanical resistance [32,49,54]. To make that happen, one might add phosphate buffer or lower the temperature. Alginate’s gelation rate might be slowed by adding cryoprotectants such as tetrasodium pyrophosphate and di- or trisodium phosphate. ALG also turns into hydrogels after rehydration [32,49,54].

Porous 3D hydrogel calcium alginate (Ca ALG) has great swelling capacity in wounds, providing slow drug release, and it is used to entrap cells for tissue regeneration and engineering, as a physical support for cells or tissue or as a hurdle between two media, because it protects the cells from the host’s immune system until it reaches the targeted area. A great example is represented by the encapsulated fibroblasts into a dual-layered structure made from alginate hydrogel with apical keratinocytes [32,58,59]. Also, a hydrogel film based on poly (N-vinyl caprolactam)-calcium alginate (PVCL/PV-Ca ALG) loaded with thrombin receptor agonist peptide (TRAP) has shown a beneficial effect on wound healing and tissue regeneration [11].

A relatively recent study compared a sodium alginate-acacia gum-based hydrogel loaded with zinc oxide nanoparticles (ZnO-NPs) to only ZnO-NPs by their healing effects and activity against *B. cereus* and *P. aeruginosa*. The authors have started from the premise that zinc helps wound healing by having antipathogenic properties, helping reepithelization and reducing the inflammation and bacterial growth in leg ulcers. This study used sheep fibroblasts and concluded that the hydrogel had less cytotoxicity than the use of only zinc oxide nanoparticles, if the concentration is carefully monitored. The hydrogel also demonstrated better results against both aforementioned rod-shaped bacteria. A complete new monolayer was observed if the plate was treated with the hydrogel, whereas the same concentration of only the nanoparticles led to cell death [29]. Also, Neacsu et al. [60] mention a study by Mohandas et al. [61] that concluded that the use of ZnO-NPs in an alginate hydrogel did have antibacterial effects against *E. coli* and *S. aureus*, but their used concentration had potentially cytotoxic effects.

When a Na ALG, chitin/chitosan, and fucoidan (60:20:2:4 *w*/*w*) hydrogel sheet (ACF-HS) was applied on rats with full thickness wounds in an in vivo cytotoxicity assay study, it provided a moist wound environment, showing easy application and removal, and enhanced cell migration [42,62,63,64,65,66]. The study involved Sprague Dawley rats treated with mitomycin C (wound-healing inhibitor) or Kaltostat^®^ (alginate-based fiber, for the positive control) and ACF hydrogel sheets were applied before being sealed with a plastic sheet [63]. Because the wounds exuded heavily the alginate-chitosan/chitin-fucoidan hydrogel sheets were replaced on day 3. The dressings were removed on day 7 and the established observation period was 18 days. The ACF-HS treated wounds displayed better healing, based on histological examinations. The wound closure and contraction, granulation, capillary formation and re-epithelization started with day 7, whether or not the wound was previously treated with mitomycin C, and the latter process was enhanced after the dressing was removed from the inhibited-healing wound, making ACF-HS a good candidate for wounds with impaired healing [63,64]. The recently developed alginate-based hydrogels are summarized in Table 1.

## 5. Alginate-Based Beads and Microcapsules for Wound Healing

The microcapsules (around 200 µm) and beads are obtained either by using CaCl_2_ as a cross-linking agent, or they can be obtained by dripping a liquid polysaccharide solution in an acidic (pH < 4) gelling solvent [15]. The bead size is also influenced by the gravity force and the resisting interfacial tension force when the droplet is falling in the liquid [45]. Furthermore, alginate beads [79], obtained through emulsion or extrusion, have the ability to entrap drugs, proteins, growth factors such as the platelet-derived growth factor (PDGF) and/or other wound healing promotors. One example is the alginate-chitosan polyelectrolyte membranes, with or without silver sulfadiazine (AgSD), and the chitosan–fibrin–sodium alginate hydrogel that displayed wound healing properties [11], as seen in Table 2.

## 6. Alginate-Based Nanofibers and Fibers for Wound Healing

When discussing the average diameter of alginate-based nanofibers obtained after electro-spinning authors mention a myriad of dimensions, ranging from 70 to almost 200 nm, as follows: 93 ± 22 nm after lavender oil was added to a Na ALG-Polyvinyl alcohol (PVA) blend; 100.35 ± 12.79 nm for a Na ALG-PVA; 105 nm for Na ALG—polyethylene oxide/glycol (PEO) blend, 175 ± 75 nm Na ALG-PVA-moxifloxacin hydrochloride [85]; 151 ± 19 nm for Na ALG-PEO [86]; 190–240 nm Na Alg-PVA [87]; 196.4 nm for a collagen-alginate [88]. The alginate-based fibers can be obtained either by spinning in an aqueous media or by extrusion. The average diameter of these fibers depends on the gauge of the used extrusion device, ranging from 70 µm up to 0.1 mm for the extruded ones, whereas fibers obtained in a coagulation bath had a diameter of 6 mm [89]. Liao et al. [90] mentions fibers with an average diameter of 10–20 µm.

A bio-polymeric system, effective in chronic wound therapy, remains a challenge. Bioactive functionalized bio-polymeric supports based on nanofibers, with integrated antibacterial components, is an area of extremely high interest, both in chemical and biopharmaceutical terms. This is because the changes in nanofibers diameters affect the rate of controlled release of the active agent within the nanofibers network [91].

When the alginate fiber dressings make contact with a wound, the space in-between its fibers will close and the bacteria will be trapped in this wound dressing because of the water intake and thus the fiber swelling [34].

**Figure 3:**
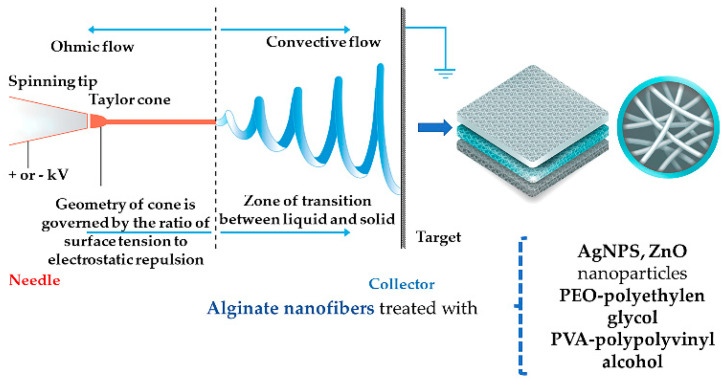
Electrospun ALG nanofibers.

Alginate-based nanofibers are obtained through electrospinning. This process takes place after high voltage electrical current passes through a liquid drop that becomes charged, and because of the antagonistic tension surface and electrostatic forces, the drop will elongate until it reaches the nanofiber state [92] (Figure 3).

The molecular weight and viscosity of the ingredients influence the resulting nanofibers [23]. Sodium alginate (Na ALG) cannot form electrospun nanofibers on its own [86,87] but the possibilities regarding the blends are numerous (Table 3). If Na ALG is blended with PEO or PVA, then the resulting nanofibers are smooth [86,93,94] and ZnO nanoparticles can be cross-linked to the mat for an antibacterial effect. When a 1:1 ratio of PVA and Na ALG was used, under a 17 kV treatment at a 0.1 mL/h rate with the collector being 5 cm away from the needle, the resulting average diameter of the nanofibers was 190–240 nm [87]. Alginate fibers can also be treated with silver nanoparticles (AgNPs) either from a silver nitrate solution or Ag^+^/Ag^0^ ions and have antimicrobial and antifungal properties [34].

PVA-Na ALG nanofibers were also generated through electrospinning at 15 kV, a flow rate of 0.5 mL/h and 15 cm away from the syringe tip and ciprofloxacin was incorporated in the created patch through active loading. The diameter of the resulting PVA-Na ALG nanofibers was 200–300 nm and it increased after the drug was loaded. These drug-loaded nanofibers showed encouraging results in an in vivo wound healing study when applied as a composite nanofiber patch following the Higuchi and Korsmeyer–Peppas drug-releasing models [95].

During an in vitro study PEO and Na ALG were used to create nanofibers at a 1:1 ratio. By adding DMSO and Triton X-100 the surface tension and the viscosity of the solution lowered. The conditions for obtaining nanofibers with an average diameter of 151 ± 19 nm were 20 kV through an 18G needle, the collector being 20 cm away from the source in a 30% humidity area. If cross-linkers like 1M calcium nitrate tetrahydrate (Ca(NO_3_)_2_) and glutaraldehyde (C_5_H_8_O_2_) treat the fibers they become thinner (Ca(NO_3_)_2_ average diameter of 149 ± 69 nm versus C_5_H_8_O_2_ average diameter 130 ± 51 nm), have better tensile strength and degradation rate, while losing their flexibility. The addition of 0.1 vol % poly-L-lysine increased the fibroblast cell attachment and their proliferation, showing the feasibility of using this type of nanofibers in other wound healing studies [86,94].

In another study, performed by Kataria et al. [95], 0.5 cm deep and 4 cm^2^ incisions were inflicted on male rabbits in a study that compared the wound healing ability of ciprofloxacin loaded- and non-loaded PVA with or without Na ALG electrospun composite nanofiber transdermal patches. The changes of the wound site were observed every 5 days for a total of 20 days and the best results were recorded the used transdermal patch was the drug-loaded PVA-Na ALG dressing. This result was proven after both histological and biochemical assays, when the complete healing of the wounded area was seen after 17 days, and the maximum amounts of collagen and hydroxyproline in the wound bed were measured after 20 days. Another advantage of the alginate-based dressing was its ability to be removed by dissolution because of its gel-forming property.

**Table 3:** Alginate-based nanofibers and fibers used for wound healing.

Composition	Study Type/Target	Ref.
2% Na ALG solution—16% PVA solution—0.5, 1, 2, 5% ZnO-NPs; Na ALG/PVA ratio 1:1	Composite nanofiber characterization, antibacterial effect, cell adhesion potential, cytotoxicity	[11,87]
Na ALG from unmodified methacrylated ALG—1% *w*/*v* RGD-modified methacrylated ALG—methacrylated heparin—4% *w*/*v* PEO; Na ALG/PEO ratio 1:1	Nanofiber characterization, cell interaction, adhesion and proliferation, binding and releasing heparin tests	[28,96]
4 wt % Na ALG—5 wt % PEO—0.5 wt % Triton X-100—5 wt % DMSO; Na ALG/PEO ratios: 65:35, 50:50, 35:65	Nanofiber characterization, fibroblast proliferation	[86,94]
8 wt % PVA—2% *w*/*w* Na ALG—3.2% *w*/*v* ciprofloxacin; PVA/Na ALG ratio: 5.5:1	Nanofiber characterization, swelling, drug incorporation and release; in vivo tests on rabbits: drug release, wound healing	[94,95]
1, 2, 3% wt of Na ALG—PEO—0.3 wt % Lecithin—5% *w*/*w* CaCl_2_; 1, 2, 3% wt of Na ALG/PEO weight % ratios: 1:1, 2:1, 3:1, 1:2, 2:2, 3:2	Solution characterization: viscosity, conductivity; Nanofiber characterization: structure, water absorption, fibroblast attachment	[93,94]
6% *w*/*v* Na ALG—0.1, 0.15, 2% chitin whisker Chitin whisker/ALG weight ratio: 0.05–2%	Whiskers and fibers characterization	[62,65,97]
ZnCl_2_—Ca ALG fibers	Antimicrobial and immuno-modulatory effects for wound healing keratinocyte migration, ppm Zn release	[11,42,89,98,99]
AgNO_3_—6% Ca ALG fibers	Antimicrobial effect	[11,89,98,100]
0.5–0.75% *w*/*v* Chitosan—0.5–1% *w*/*v* ALG—Dexamethasone/BSA/PDGF-bb/Avidin fibers	Drug incorporation and release, PDGF-bb bioactivity	[49,90]
0, 0.014, 0.041% *w*/*v* Chitosan—0.001% *w*/*v* Na ALG—Ninhydrin—CaCl_2_ in fibers	Filament characterization	[49,101]
1.5 w % Na ALG—Ag-NPs in crosslinked fibers	Wound healing effect on SKH-1 mice	[102]

An uniform morphology of the nanofibers can also be obtained by adding lecithin as a natural surfactant [93], or arginine–glycine–aspartic acid (RGD) [103].

Nanofibers can also be obtained by mixing methacrylated alginate, RGD-modified methacrylated alginate and PEO at 10.4 kV, with a flow rate of 0.6 mL/h and the collector being placed at 15 cm away from the syringe tip. The interesting part about this blend was the UV treatment with 365 nm UV light at <1 mW/cm^2^ that can or cannot be followed by the PEO extraction. The resulting photo-cross-linked nanofibers can also be coated with gold. Before the cross-linking the fiber diameters were between 185.5 ± 37 and 195.4 ± 23 nm, after cross-linking the fiber diameters were between 182.2 ± 36 and 190.4 ± 30 nm, but the PEO extraction lead to a diameter increase due to nanofiber swelling, ranging between 256.3 ± 43 and 297.9 ± 42 nm [96]. When this study used PEO/methacrylated heparin-, RGD-modified-, or unmodified methacrylated alginate-based nanofibers, it concluded that—by adding methacrylated heparin—the stress–strain curves are influenced, therefore making the elongation at break significantly lower and the tensile strength and Young’s modulus significantly greater than those observed for the unmodified or RGD-modified methacrylated alginate-based nanofibers [96].

## 7. Other Alginate-Based Dressings

Among the alginate-based wound healing blends (Table 4) are gelatin-alginate sponges, alginate-scaffolds based on antisense oligo-deoxynucleotides (asODN) linking to Connexin 43 (Cx43) [11,104], viscose/Ag NPs/ALG/nicotinamide/CaCl_2_ fabrics [105]. The 3D bi-layered scaffold made of polyethylene glycol (PEG)-chitosan hydrogel and chitosan-alginate can also help tissue regeneration after injury by holding fibroblasts on the upper surface and keratinocytes on the lower one [106,107].

The wound pH is a reliable factor when discussion the healing status, because it shifts from high when infected to low when healed either naturally or because it was modulated through applying different treatments [108,109,110]. Polyethylene oxide–alginate wafers loaded with diclofenac and streptomycin show controlled drug release at room temperature, in simulated wound fluid (BSA, CaCl_2_, NaCl, C_4_H_11_NO_3_) conditions at pH 7.5. The diffusion of both drugs from the annealed wafers takes place slowly, making them potentially useful in highly exuding wounds [23,111]. Because wound pH-variation has a strong effect on the healing process, researchers also developed, through microfluidic spinning using their electrostatic interactions, a mesoporous particle hydrogel alginate-based flexible microfiber linked to a pH-responsive dye linked onto a transparent medical tape place on top of a wound, in order to observe the pH modifications in real-time [112].

A 3D porous sponge was obtained after a pre-gelled (with bivalent cations) alginate was frozen and then lyophilized. The type and concentration of both alginate and cross-linkers, as well as the freezing protocol influenced the mechanical properties as well as the size (70–300 μm) and display pattern of the pores. This pore size was appropriate for fibroblast seeding [113]. On the other hand, when comparing the tensile strength for G-ALG and M-ALG sponge dressings with different ALG concentrations, the elongation at fracture was not influenced by the alginate concentration, whereas Young’s modulus and the maximum stress at fracture increased with it [114].

**Table 4:** Other alginate-based dressings used for wound healing.

Composition	Study Type/Target	Ref.
Na ALG—0.1% *w*/*v* I-labeled SDF-1 plasmid Na ALG—0.0001% *w*/*v* (1 ng/µL) I-labeled SDF-1 protein	Acute surgical wounds on Yorkshire pigs: SDF-1 release kinetics, wound healing rate, scar formation	[42,115]
Viscose/silver/ALG Vis/Ag-NPs/ALG Viscose/silver/ALG/nicotinamide Viscose/silver/ALG/nicotinamide/CaCl_2_ 0.5–1.5% *w*/*w* Na ALG in nonwoven fabric	Burn—diabetic rats	[105]
Chitosan 4% *w*/*v*—1% *w*/*w* CH_3_COOH—4% ALG Chitosan 4% *w*/*v*—1% *w*/*w* CH_3_COOH—4% CG *w*/*v*—5.7% NaCl	Comparative drug release system study: swelling, Diltiazem HCl-loaded tablet formulation, dissolution in 1:1 complex systems	[116]
Ca ALG versus silicone-coated polyamide net	Comparative randomized trial: healing and slippage rate, removal discomfort degree on skin graft donor sites	[117,118]
2% *w*/*v* Na ALG—pH-responsive dye—glycerol 0–60% *w*/*v*	Flexible microfibers description, real-time pH modifications on the pig wound site	[112]
collagen—0.5, 1.0, 1.5, 2.0, 3.0, 4.0, 5.0% alginic acid, at 3:1 ratio in cross-linked sheet	Sheet characterization	[98,119]
75% Alginic acid solution 2% *w*/*v*– 45S5 bioactive glass—25% cell suspension	VEGF secretion, cell viability, cytotoxicity	[120]
Polyox^®^—CG—Streptomycin—Diclofenac (0.75:0.25:0.3:0.25 g) Polyox^®^—0.5 g Na ALG—Streptomycin—Diclofenac (0.5:0.5:0.25:0.1 g)	Wafer characterization, adhesion, drug release, swelling	[23,111]
2% *w*/*v* Alginic acid—murine antisense nucleotides (Cx43asODN) based scaffolds	Wounded ICR mice: inflammatory response, re-epithelization	[11,104]
20 µg Smad3 ASOs in Na ALG and chitosan 1:1, 1:2, 1:4, 1:8, 1:16 ALG/chitosan ratio in PEC	Scaffold characterization, wound healing in C57BL/6 mice	[40,62,65]
gelatin—1 wt % Na ALG (with or without 0.4 mg/cm^2^ AgSD) sponge	Wistar rat wound healing	[11,98,121]
1% solution silk fibroin—1% solution alginic acid in 10:0, 5:5 and 0:10 ratios sponge	Sprague Dawley rats full thickness wound	[98,122]
2 or 4% *w*/*v* high G and high M ALG—0.1–10% *w*/*w* PEG—9.5% *w*/*v* poly(D,L-lactide-*co*-glycolide) (PLGA)—0.5% *w*/*v* insulin microparticles in sponge	Sponge characterization: density, tensile strength, water vapor transmission rate and absorption capacity. In vitro study: interaction with HaCaT cells, insulin release	[23,114]
Chitosan—1% *w*/*w* Na ALG—hematoxylin-eosin—DHEA—AgSD in PEC sponge	Microscope assay, in vitro drug release, cytotoxicity, antibacterial effect, in vivo burn healing on BALB/C mice	[49,123]
ALG—0.2, 0.4% chitosan—0.1, 0.5% all-trans retinoic acid (ATRA). 1:10 ATRA/ALG ratio in microparticles	Microparticles characterization, encapsulation efficiency, dermal localization, ATRA skin release	[42,124]
2% Na ALG (61% M and 39% G)—2–4 µg VEGF in microspheres	In vitro drug release, in vivo angiogenesis	[125]

PEG addition to the aforementioned sponge increases the flexibility while having a plasticizing effect. Furthermore, its concentration and molecular weight significantly modifies the tensile strength of the sponge. For low molecular weight PEG of 1.45 kDa a concentration increases from 0.1% to 1% leads to a lower Young’s modulus, maximum stress at fracture and elongation at fracture. For high molecular weight PEG (10 kDa) the concentration increases from 0.1% to 1% leads to an increase for Young’s modulus and maximum stress at fracture, while the elongation at fracture is significantly lower. While comparing the results between the uses of different molecular weight PEG in the sponge, the one with a molecular weight of 1.45 kDa displayed higher tensile strength values than the 10 kDa PEG. PEG addition to the M-ALG sponges also increases the WVTR and decreases the water absorption capacity, regardless of its molecular weight, but the observed values for WVTR (16.7 ± 0.4 before PEG addition and (22.9 ± 0.8)–(28.8 ± 2.1) after PEG addition) were almost double the recommended values (8–10 mg/cm^2^/h) [114].

## 8. Commercially Available Pharmaceutical Alginate-Based Products

The list of commercially available alginate-based wound dressing is growing fast (Table 5), while the most recent FDA approval available on-line at this time was given to Luofucon^®^ Extra Silver Alginate Dressing (Prescribed only—PO) and Luofucon^®^ Antibacterial Alginate Wound Dressing (Over-The-Counter—OTC) [126,127]. The researchers state the dressings proved their activity against *E. coli*, *E. faecalis*, *K. pneumoniae*, Methicillin-resistant *S. aureus* (MRSA), *P. aeruginosa*, *S. aureus*, *S. pyogenes*, and Vancomycin-resistant *Enterococcus* (VRE). Silver creates a barrier against a broad spectrum of bacteria [128] for as long as seven days [126].

Enzymes were added into a PEG-alginate hydrogel called Flaminal^®^ Forte that underwent human clinical trials for treating partial thickness burns [129] and had better results than the Ag sulfadiazine-based one (Flamazine^®^), regarding the times it needed to be changed [130]. When a patient is unable to move, pressure ulcers may appear. Such a case was also healed after several dressings were applied, including a honey-loaded alginate-based product, named Algivon^®^ [131,132]. An improved version of this dressing, Algivon^®^ Plus, showed good clinical results when applied on chronic wounds [133]. Algivon^®^ Ribbon or Plus is also used for diabetic, pressure, and leg ulcers, fungating lesions, infected, cavity, and chronic or complicated surgical wounds and abrasions [32,131,133,134,135,136]. On the other hand, another Manuka honey loaded calcium alginate-based wound dressing is Activon^®^ Tube or Tulle for diabetic and leg ulcers, pressure sores, and malodorous, infected, dry, sloughy, or necrotic wounds [135,137,138,139].

Recently, researchers have studied the effect of treating human skin lesions, produced by an atypical form of Henoch–Shönlein purpura, with three hyaluronic acid-based commercially available products Hyalomatrix PA^®^ (a 3D matrix of a hyaluronic acid ester (Hyaff) and a transparent film), Hyalogran^®^, and Jaloskin^®^, which were maintained on the wound site for various time periods in a consecutive order. One of the treatments, Hyalogran^®^, was an alginate-hyaluronan dressing made of sodium alginate and Hyaff [140]. After the eschar resection, wound debridement, and 21 days of the first dressing, the next step was applying the second one for two weeks, and the third for an unmentioned period. After two months from the first treatment the wound was completely healed [140], with minimal scaring and thus confirming the benefits of wound treatments involving hyaluronic acid in combination with sodium alginate. Silvercel^®^, another commercially available alginate-based non-adherent dressing, was used in a wound healing study, when it was applied twice a week on a diabetic patient with a repetitive non-infected venous leg ulcer and the wound healed in 14 day [141].

**Table 5:** Alginate-based commercially available pharmaceutical products used in wound healing.

Name	Composition/Target	Study Type/Effects	Ref.
ALGS6 Ag (Prescribed/Over-The-Counter)	Ca ALG fiber—Lyocell fiber—1.7% Ag^+^ Surgical, traumatic, acute and chronic wounds, ulcers, first and second degree burns; Minor cuts, burns	Exudate absorption, gel forming, wound healing promoter if changed weekly	[142]
Aquacel™ Ag EXTRA™ Hydrofiber™ (Prescribed/OTC)	Bi-layer Na ALG CMC—Ag^+^—strengthening fibers; Surgical, traumatic, exuding, infected, and painful wounds, second degree burns, ulcers or for minor cuts or burns	Bacterial inhibition, absorbs the wound exudate while forming a gel, it must be changed every 1–2 weeks	[142]
Calgitrol™ foam or SilverSite™	Ag+ in Ag ALG—Ca ALG—polyurethane foam	Antimicrobial effect, cytotoxicity	[23,143,144,145]
Fibracol 10% Ca ALG in Fibracol Plus^®^ dressing	90% Collagen—10% Ca ALG; Exuding full- and partial-thickness wounds, second degree burns; diabetic, pressure, and venous ulcers	Clinical Trial: healing time of diabetic foot ulcers	[16,32,146]
Flaminal^®^ gel	ALG—PEG matrix—notatin—lactoperoxidase—guaiacol; burns, post-surgery wounds, diabetic, leg and pressure ulcers. If used with H_2_O_2_ and SCN- it has a bacteriostatic effect against both gram-positive and gram-negative bacteria.	Antimicrobial and bacteriostatic effect, wound surface moisturizer, exudate absorber, debrides necrotic tissue	[32,129,147,148]
Guardix-SG^®^	Na ALG—poloxamer—CaCl_2_; post-surgery (silicone implantation, blepharoplasty)	In vivo post-surgery studies on rabbits: thermosensitive gel, mechanical barrier formation, suppression of capsular contracture, reduced inflammation and fibrosis	[32,149,150]
Hyalogran^®^ dressing	Hyaluronic acid ester—Na ALG; Leg and pressure sores, diabetic and ischemic wounds (with slough or necrosis)	Exudate absorption, gel transformation, necrotic tissue removal	[32,140]
Kaltostat^®^	80% High G Ca ALG—20% NaALG; Acute and chronic wounds with moderate to heavy exudate	Wound healing	[6,28,49,62,63,64,65,151]
Luofucon^®^ Antibacterial Alginate Wound Dressing (OTC)	Ca ALG—Ag; minor cuts, abrasions, and burns	Changed daily it has antibacterial effect and promotes wound healing	[126]
Luofucon^®^ Extra Silver Alginate Dressing (PO)	Ca ALG—Ag; moderate to heavily exuding wounds, ulcers, trauma-inflicted or post-operative wounds, infected wounds, grafts	Antibacterial effect for seven days and it promotes wound healing	[126]
Medihoney^®^ (gel, hydrogel or paste)	Ca ALG—Manuka honey; ulcers: hemorrhagic, heavily exuding, diabetic foot, leg (arterial or venous), pressure (partial or full-thickness) ulcers. First and second partial thickness burns. Surgical and traumatic wounds	Antibacterial effect	[3,23]
Purilon Gel^®^	Na CMC—Ca ALG; Used with another dressing for first and second degree burns or sloughy and necrotic wounds	Wound surface moisturizer	[32,147,152,153]
Saf-gel^®^	Carbomer propylene glycol sodium—Ca ALG; Abrasions, cuts, sloughy and necrotic wounds, second degree burns, non-infected diabetic foot ulcers, and pressure and venous ulcers	Wound healing and surface moisturizer.	[32,147,154]
SeaSorb^®^ fine foam sheet	Na ALG/Ca ALG—polyethylene net; Heavily exuding wounds: cavity wounds, second degree burns, diabetic, leg and pressure ulcers, spina bifida	Human clinical trials: fiber to gel transformation, tolerance, healing rate with reduced exudate, maceration and pain intensity	[32,155,156]
Silvercel™	36% Ca ALG with high G—6% CMC—28% Ag (111 mg Ag/100 cm^2^)—30% EasyLift Precision Film (Acelity/Systagenix)	Pig and human trials, wound healing	[141,157,158,159,160,161,162]
Tromboguard^®^ bi-layer dressing	Polyurethane sponge—chitosan + Na ALG + Ca ALG + Ag^+^; Traumatic and post-surgery wounds	Antibacterial effect and strong hemostatic	[32]

Other commercially available dressings contain both ALG and silver, like Aquacel™ Ag EXTRA™ Hydrofiber™ or ALGS6 Ag Alginate Wound Dressing have similar wound healing characteristics [142]. Various other commercially available alginate-based dressings that were included in a multiple comparative studies were Suprasorb^®^ A (100% ALG), Suprasorb^®^ A—Ag (ALG—ionic Ag), and LG—nano Ag ^®^ Acticoat Absorbent with SILCRYST™, for their anti-microbial effect, binding to elastase capacity, MMP-2, TNF-α and IL-8, antioxidant ability, cytotoxicity, and effect on HaCaT keratinocytes, showing promising results [42,163,164].

## 9. Conclusions

The development of alginate-based biomaterials for wound healing has an accelerated pace. The last years gave patients hopes for receiving better treatment for their wounds because the development of a wound dressing that might actually become a true ‘ideal dressing’ seems to be in hands reach. The versatility of alginate-based wound dressings, the promising results after both in vivo and in vitro trials and the cost-effectiveness of obtaining them makes alginate one of the favorites when choosing the material that could act both as a support and as a carrier for the bio-active compounds that have to reach a wound.

## Figures and Tables

**Table 1:** Alginate-based hydrogels used for wound healing.

Composition	Study Type/Target	Ref.
1% *w*/*v* Na ALG—0.1% *w*/*v* acacia gum—1 mg/mL ZnO-NPs	Characterization, healing effects and cytotoxicity on sheep fibroblasts, antibacterial activity	[29]
0.5–2.5% *w*/*v* LF 200S ALG hydrogel emulsion + 0.9, 1.4, 2.8% Tween 80/Span 20/isopropyl myristate oil/Ketoprofen ratio 26:1.25:4:1 Ca^2+^/D (+) gluconic acid δ-lactone molar ratio: 1:2 Ketoprofen microemulsion: 0.9, 1.4, 2.8%	Characterization, drug release, scattering patterns	[56]
alginate dialdehyde (ADA)—gelatin—0.1 M borax	Characterization and in vivo study on rat model	[57]
60:20:2:4 *w*/*w* Na ALG—chitin/chitosan—fucoidan hydrogel sheet	Sprague-Dawley rats with full thickness wounds, gives a moist wound environment, easy application and removal, migration, cytotoxicity assay	[62,63,64,65,66]
encapsulated TRAP—0.5% *w*/*w* chitosan—PV-Ca ALG hydrogel film	C57 black 6/CBA mice wound healing	[67]
10 g ALG—4 mg trypan blue, 10 g ALG—10 µg VEGF	Drug release: in vitro and in vivo on NOD and SCID mice, angiogenesis	[68]
1% heparin—1% Na ALG—bFGF	Characterization, angiogenesis and bFGF release profile in Wistar rats	[69]
10 & 15% Polyvinyl pyrrolidone—0.5 and 1% ALG—0, 30, 50, 70, and 100 ppm Ag-NPs.	Characterization, crosslinking degree, antimicrobial activity. Best results: 0.5% ALG, 15% PVP, 70 ppm Ag-NPs	[48]
Polyurethane foam—pH-sensitive Na ALG-bentonite hydrogels 1:0, 0.7:0.3, 0.5:0.5, 0.3:0.7	Characterization, drug release from foam, cytotoxicity	[70]
Micro-emulsion 20% Tea tree oil—1% *w*/*v* ALG hydrogel	Characterization, oil dispersion, antimicrobial effect	[71]
10% Na ALG sulfate—CM11 peptide	Mouse wound healing	[72]
ALG—k-CG ratio: 5:5, 7:3, 8:2 ALG—i-CG ratio: 5:5, 7:3, 8:2	Formation and characterization; cytotoxicity, cell encapsulation	[73]
VEGF—2 wt % Na ALG, VEGF—0.05% chitosan—2 wt % Na ALG VEGF—heparin-coated chitosan—2 wt % Na ALG	In vitro drug release	[74]
2 wt % ALG—trypan blue, 2 wt % ALG—methylene blue, 2 wt % ALG—VEGF	In vitro drug release	[75,76]
PEG diacrylate—thiolated ALG bilayered hydrogel with small extracellular vesicles (sEVs)	Characterization, rats and rabbit full thickness wound size reduction, sEVs release, angiogenesis, collagen arrangement	[77]
Sr^2+^ loaded Na ALG aldehyde—polyetherimide (PEI)	Characterization, hydrogel self-healing behavior, in vitro cell response, cytotoxicity, rat wound healing	[78]

**Table 2:** Alginate-based beads and microcapsules used for wound healing.

Composition	Study Type/Target	Ref.
3.5% Na ALG—3% KCl—3.5% k-CG—3% CaCl_2_ Na ALG/k-CG weight ratio: 10:0, 9:1, 8:2, 7:3, 6:4, 5:5, 4:6, 3:7, 2:8, 1:9, 0:10	Characterization and thermostability	[80]
ALG—k-CG ratio: 5:5, 7:3, 8:2 ALG—i-CG ratio: 5:5, 7:3, 8:2	Formation and characterization; cytotoxicity, cell encapsulation	[73]
3% *w*/*v* diclofenac—1–3% *w*/*v* Na CMC—0.5% *w*/*v* AlCl_3_ 6H_2_O 3% *w*/*v* diclofenac—1–3% *w*/*v* Na ALG—5% *w*/*v* AlCl_3_	Drug content and particle size, disintegration, friability and in vitro dissolution test, in vivo test on beagles	[50,81]
Na ALG/k-CG %: 100, 75:25, 50:50, 25:75 + 0.125 g Fe_3_O_4_	Hydrogel magnetic beads characterization, drug release, swelling	[82]
2% *w*/*v* high M Na ALG—VEGF	In vitro drug analysis	[83]
Beads of 1% Ca ALG—0.25% platelet lysate—0.03% vancomycin hydrochloride	Particle characterization, drug and PDGF AB release, PBS absorption, cell proliferation	[84]
